# Don’t believe them! Reducing misinformation influence through source discreditation

**DOI:** 10.1186/s41235-024-00581-7

**Published:** 2024-08-26

**Authors:** Ullrich K. H. Ecker, Toby Prike, Antonia B. Paver, Rosie J. Scott, Briony Swire-Thompson

**Affiliations:** 1 School of Psychological Science, University of Western Australia (M304), Perth, 6009 Australia; 2 Public Policy Institute, University of Western Australia, Perth, Australia; 3 School of Psychology, University of Adelaide, Adelaide, Australia; 4 College of Social Sciences and Humanities, Northeastern University, Boston, USA

**Keywords:** Misinformation, Disinformation, Continued influence effect, Source discreditation, Derogation, Debunking

## Abstract

Misinformation often continues to influence people’s reasoning even after it has been corrected. Therefore, an important aim of applied cognition research is to identify effective measures to counter misinformation. One frequently recommended but hitherto insufficiently tested strategy is source discreditation, that is, attacking the credibility of a misinformation source. In two experiments, we tested whether immediate source discreditation could reduce people’s subsequent reliance on fictional event-related misinformation. In Experiment 1, the discreditation targeted a person source of misinformation, pointing to a conflict of interest. This intervention was compared with a commonly employed message-focused correction and a combination of correction and discreditation. The discreditation alone was effective, but less effective than a correction, with the combination of both most effective. Experiment 2 compared discreditations that targeted a person versus a media source of misinformation, pointing either to a conflict of interest or a poor track record of communication. Discreditations were effective for both types of sources, although track-record discreditations were less effective when the misinformation source was a media outlet compared to a person. Results demonstrate that continued influence of misinformation is shaped by social as well as cognitive factors and that source discreditation is a broadly applicable misinformation countermeasure.

## Introduction

Misinformation can lead people to form false beliefs and distort their understanding of events (Ecker et al., 2022). When misinformation relates to important domains such as health, politics, or science, it can be harmful at a societal level (Bennett & Livingston, 2018; Bursztyn et al., 2020; Lewandowsky et al., 2017, 2023, 2024; Loomba et al., 2021; Poland & Spier, 2010; Simonov et al., 2022; Swire-Thompson & Lazer, 2020; Tay et al., 2024; van der Linden et al., 2017). Therefore, finding the most effective strategies to counter misinformation is an important focus of contemporary research (Ecker et al., 2022; Ha et al., 2021; Kozyreva et al., 2022; Ziemer & Rothmund, 2024). One commonly recommended but under-researched approach is to discredit misinformation sources (Lewandowsky et al., 2020; Paynter et al., 2019). Here, we examine the efficacy of source discreditation in countering misinformation and contrast it with the well-established approach of debunking (see Prike & Ecker, 2023 for a review).

While pre-emptive misinformation interventions are increasingly being explored—such as those that aim to equip information consumers with the skills to identify misleading argumentation in order to inoculate them against future misdirection (Cook et al., 2017; Lewandowsky & van der Linden, 2021)—the retrospective correction (or “debunking”) of false claims is still an important tool for fact-checkers and communicators if the first line of defense is not implemented or successful (Prike & Ecker, 2023). Debunking is also generally more effective at reducing belief in specific claims than pre-emptive skill-based interventions, making it particularly important for countering harmful misinformation (Chan & Albarracín, 2023; Walter et al., 2020). A pervasive challenge for debunking interventions is the robust finding that even after misinformation has been credibly corrected, people often continue to rely on it in their thinking and reasoning—a phenomenon known as the continued influence effect (Chan et al., 2017; Ecker et al., 2022; Johnson & Seifert, 1994; Walter & Tukachinsky, 2020). Continued influence effects may be particularly pronounced with delayed corrections (Walter & Tukachinsky, 2020) but generally arise regardless of the interval between misinformation and correction (Ahn et al., 2023; Rich & Zaragoza, 2020). As such, the present study focused on immediate corrections, which often occur in the real world when an initial false claim is instantly rebutted in a conversation or an article presenting different viewpoints, or when a social media user reads a post and a corrective comment in direct succession.

From a theoretical perspective, continued influence has been linked to failures of memory integration and retrieval—specifically incomplete integration of corrective information within the relevant mental model (e.g., a mental event model; Bower & Morrow, 1990; Johnson-Laird, 1994), or selective retrieval of the misinformation without the associated corrective information during (event-related) reasoning (for a review, see Ecker et al., 2022). However, it is widely acknowledged that social factors also shape misinformation impacts.

One social factor known to affect reliance on corrected misinformation is source credibility, which refers to the perceived trustworthiness and expertise of a message source (Pornpitakpan, 2004). Source credibility has been found to influence persuasion through multiple different mechanisms (Briñol & Petty, 2009). For example, it can bias the cognitive evaluation of a message or affect the degree of confidence people have in their evaluation (Tormala et al., 2007). It can also serve as a heuristic, guiding acceptance or rejection of the persuasive message, especially if the recipient lacks time or motivation to engage analytically with the message (Metzger & Flanagin, 2013; Petty et al., 1981). In line with this, substantial evidence suggests that source credibility affects initial belief in true and false information (e.g., Nadarevic et al., 2020; Swire et al., 2017; Zhu et al., 2010). It is also known that the impact of retracted misinformation on people’s reasoning is diminished when they are suspicious of the misinformation source’s motives (Fein et al., 1997; Lewandowsky et al., 2005), and that credibility of the correction source influences correction effectiveness, (Amazeen & Krishna, 2023; Ecker & Antonio, 2021; Guillory & Geraci, 2013; van der Meer & Jin, 2020; Vraga & Bode, 2017, 2018; Wood et al., 2023). Source credibility also features prominently in theoretical models of continued influence that focus on the rationality of belief updating in light of new evidence. Such models propose that misinformation will have continued, post-correction influence on reasoning to the extent that the misinformation is perceived as more reliable than the correction (Connor Desai et al., 2020; also see O’Rear & Radvansky, 2020; Zmigrod et al., 2023).

Considering these findings together, explicitly discrediting a misinformation source in order to reduce its credibility has been one recommended component of best-practice debunking approaches (Lewandowsky et al., 2020; Paynter et al., 2019). Walter and Tukachinsky (2020) suggested that undermining the credibility of a misinformation source (e.g., by highlighting a conflict of interest) may assist individuals to dismiss misinformation by offering an explanation as to why the misinformation was initially provided. However, only limited research has empirically investigated the efficacy of this approach.

A study by Campos-Castillo and Shuster (2021) found that source discreditation indeed reduces perceived source credibility. However, it is important to take investigations beyond direct effects on perceived credibility to demonstrate downstream impacts on misinformation reliance. In this vein, Westbrook et al. (2023) found that not only did a correction affect the perceived credibility of a misinformation source, but adding an explicit source discreditation made a correction more effective. This is in line with a number of other investigations that included source discreditation alongside content-focused corrections as an element of a successful debunking intervention (e.g., MacFarlane et al., 2021; Paynter et al., 2019; Tay et al., 2022). A recent study conducted in a simulated social media environment found that the presence of source-credibility information boosted participants’ discernment between true and false social media posts (Prike et al., 2023). The authors argued that this effect was in part driven by a correlation between source-credibility scores and the veracity of the online post.^1^ In other words, the greater a source’s credibility score, the more accurate their information was perceived to be. In a forensic context, Lagnado and Harvey (2008) found that discrediting a crime witness led mock jurors to dismiss their evidence (also see Fein et al., 1997). The perhaps strongest evidence for the efficacy of discreditations comes from a study by Barnes et al. (2018), who found that attacking the trustworthiness of a scientist (e.g., by pointing to a conflict of interest or past data fabrication) reduced belief in misleading claims made by the scientist as much as directly challenging the veracity of the claims themselves. However, attacking the scientist’s competence (e.g., stating they were a “sloppy” researcher or had a degree from a university with low standards) had no effect.

By contrast, a recent study found that labeling a piece of misinformation as the result of either intentional deception or accidental error had no impact on correction effectiveness (Connor Desai & Reimers, 2023). Likewise, Wood et al. (2023) found that debunking COVID-19 misinformation reduced misperceptions (as long as the correction source was credible) but that directly discrediting sources of vaccine misinformation (i.e., anti-vaxxers) had no effect on participants’ misperceptions. Overall, there is therefore tentative but somewhat inconclusive evidence regarding the efficacy of source discreditation as a debunking tool, especially when used as a stand-alone intervention in the absence of a content-focused correction targeting the misinformation directly. The focus of Experiment 1 was thus to conduct a rigorous test of the effectiveness of source discreditations to reduce misinformation impacts, given the lack of solid evidence in the existing literature.

One additional consideration is that news reports are often not provided by people but by media sources, and evidence regarding source-credibility effects in media contexts is even less conclusive. On the one hand, people do take source reputation into account when evaluating media sources (Metzger & Flanagin, 2013) and tend to distrust and avoid unfamiliar or disreputable news sources (Pennycook & Rand, 2019; Peterson & Allamong, 2022). Further, some studies have found that news items are perceived as more truthful when they come from reputable news sources rather than fictitious ones (Bauer & Clemm von Hohenberg, 2021; Nadarevic et al., 2020). Kim et al. (2019) investigated the impact of labeling fictional news headlines with source reputation ratings, finding that headlines were generally less believable when attributed to sources with poor reputation (vs. unlabeled or highly rated sources). Similarly, Heinbach et al. (2018) reported that news articles are more persuasive when they come from a high- versus low-credibility media source.

On the other hand, the credibility of media sources has sometimes been found not to influence content processing (Austin & Dong, 1994; Shen et al., 2019; Sterrett et al., 2019; Tsang, 2021). For instance, Peterson and Allamong (2022) found that political news exerted similar effects on readers’ opinions regardless of whether the source was an established or an unfamiliar media outlet. In a field experiment, Aslett et al. (2022) found that badges signaling the credibility of media sources online failed to affect participants’ belief in both true and false claims from low-quality sources. Furthermore, several studies have failed to show that headline veracity judgments are affected by source information (Clayton et al., 2019; Pennycook & Rand, 2020). For example, Dias et al. (2020) demonstrated that varying the visibility of news source information alongside news headlines had no significant effect on their perceived veracity, suggesting that participants generally attended more to headline plausibility rather than source credibility. Furthermore, Wintersieck et al. (2021) found that the efficacy of fact-checks of political advertisement claims was only modestly impacted by the perceived credibility of the correction source, with the effect driven mainly by partisan congeniality, that is, the ideological congruence of the participant and the media source providing the correction.

Thus, overall, it appears that the credibility of media sources has less of an impact on the acceptance of misinformation compared to the credibility of person sources. Experiment 2 therefore replicated Experiment 1 but extended it by contrasting discreditations of person versus media sources.

## Experiment 1

Experiment 1 was designed to test whether source discreditation as a stand-alone intervention can reduce misinformation reliance. It additionally aimed to compare the effectiveness of source discreditation versus message-focused correction and to test whether a combined approach of correcting and discrediting is more powerful than either strategy in isolation (as suggested by Westbrook et al., 2023). To this end, participants were provided with fictional news reports that (in the experimental conditions) contained a critical piece of information deemed to be false. This misinformation was then either retracted, the source of the misinformation was discredited, or both, or neither. Discreditations were designed specifically to lower perceived trustworthiness, by detailing a conflict of interest, as research has suggested that trustworthiness is a primary dimension of credibility (Barnes et al., 2018; Ecker & Antonio, 2021; Guillory & Geraci, 2013; McGinnies & Ward, 1980; but also see Susmann & Wegener, 2023b). Participants’ subsequent reliance on the misinformation was measured using inferential reasoning questions about the events described in the reports.

There were two main hypotheses: It was predicted that both individual debunking interventions (correction and discreditation) would reduce misinformation reliance relative to a no-intervention (misinformation) condition (H1; in line with Barnes et al., 2018). Second, the combination of correction and source discreditation was expected to provide the greatest reduction in misinformation reliance (H2; in line with Westbrook et al., 2023; note that we expected this combined effect to be sub-additive). There were two secondary hypotheses: Corrections were expected to reduce misinformation reliance more effectively than discreditations (H3), given that a correction inevitably also subtly discredits a misinformation source to some degree (i.e., any correction implies that the source was untrustworthy at least on this occasion). Moreover, as even effective debunking strategies typically do not eliminate misinformation reliance (Ecker et al., 2010; Walter & Tukachinsky, 2020), a continued influence effect was expected relative to a no-misinformation control condition (H4).

## Method

Experiment 1 used a 2 × 2 plus control within-subjects design, with conditions relating to whether misinformation content was corrected (no/yes) and whether the misinformation source was discredited (no/yes). The control condition used materials not containing any misinformation, correction, or discreditation.

### Participants

An a priori power analysis conducted using G*Power 3 (Faul et al., 2007) indicated that a minimum sample size of 200 was required to detect a difference between two conditions of effect size *f* = 0.2 (*α* = 0.05; 1 – *β* = 0.8).^2^ To ensure ample power, a total of 300 US-based Prolific (www.prolific.com) users with a minimum platform approval rating of 95% were recruited. Eight participants were excluded based on inconsistent responding between normal and reverse-coded items, as per an a priori exclusion criterion (see Supplement for details, available at https://osf.io/vqth3/). No participants were excluded due to the additional criterion of self-rated “poor” or “fair” English proficiency (Likert scale 1 = *poor* to 5 = *excellent*). The final sample size for analysis was thus *N* = 292. The sample comprised 167 female, 119 male, and 4 non-binary participants; 2 participants did not state their gender. Age ranged from 19 to 77 years (*M* = 40.31; *SD* = 13.94).

### Materials

An example article with all test questions is provided in Table 3; all articles and test questions can be found in the Supplement.

*Articles*. Participants were presented with five fictional news articles on current affairs. Topics included menopause treatments (allegedly now including a drug initially developed for high blood pressure), construction of a new mine in Western Australia (allegedly safeguarding local Indigenous rock art), renewal of a beachside suburb (allegedly accompanied by an increasing burglary rate), a Canadian band canceling a concert (allegedly because they split up), and a London restaurant closing (allegedly due to a vermin infestation). Explicitly political topics were avoided to prevent participants’ political affiliations or beliefs from potentially impacting results.

Each article existed in five versions, corresponding to the five conditions (control, misinformation, correction, discreditation, and combined). In the no-misinformation control condition, the article contained only generic information deemed to be factual (e.g., descriptions of menopausal symptoms and treatments). In the misinformation condition, the article included information deemed false, which was not challenged (e.g., a health researcher claiming that “a drug called Cendexyl, initially developed to treat low blood pressure, has also been found to be an effective treatment for menopause symptoms”). The correction condition included the misinformation plus a statement from a seemingly knowledgeable source correcting it (e.g., a gynecologist stating that it is incorrect that Cendexyl is an effective treatment for menopause symptoms and that “based on the overall evidence available, this is just entirely false”). In the discreditation condition, the misinformation was followed by a statement accusing the misinformation source of having vested interests (e.g., the gynecologist explaining that there was a conflict of interest because the researcher did not disclose funding by the pharmaceutical company behind Cendexyl). The discreditation stimuli were pilot tested to establish that they affected perceived source credibility (see Supplement for details). The combined condition included the misinformation plus both the correction and the source discreditation. All interventions also stated that the initial information provided should be disregarded. The assignment of articles to conditions and the presentation order of articles and conditions were counterbalanced using a Graeco-Latin square; thus, participants were randomly allocated to one of five survey versions, and each participant read five articles—one per condition.

*Test questionnaire*. To measure how much participants relied on the misinformation in their reasoning, they were given a questionnaire that featured a set of questions relating to each article (see Supplement). Each question set comprised five inferential reasoning questions; these were presented individually and used 11-point Likert scales (e.g., “Cendexyl treatments should be covered by health insurance for menopausal symptoms”—“Strongly Disagree” [0] to “Strongly Agree” [10]); two questions per question set were reverse coded (e.g., “Doctors should refrain from prescribing Cendexyl for menopause.”—“strongly disagree” [0] to “strongly agree” [10]). All test questions were presented for a minimum of 3 s before participants were able to proceed.

### Procedure

The survey was built with Qualtrics (Qualtrics, Provo, UT, USA). Participants were first presented with an ethics-approved information sheet and provided informed consent by ticking a box in the online survey. Participants then received the five articles in an order determined by the randomly allocated survey version. Reading was self-paced, but each article was presented for a minimum time (set at approximately 150 ms per word). Next, participants completed a brief 1-min distractor task (a word puzzle) and then answered the questionnaire, with the order of the five question sets corresponding to the sequence of articles. Prior to completing each questionnaire, participants were provided with a short recap of the article content (e.g., “Think back to the article you read on menopause treatments while completing the following questions.”). All participants were provided with a debriefing sheet emphasizing that the articles were entirely fictitious and explaining the design, purpose, and potential benefits of the experiment (following best-practice guidelines; Greene et al., 2023). Median completion time was 10 min; participants were paid £1.35 (approximately US$1.56).

## Results

Prior to conducting any analyses, scores associated with reverse-coded questions were reverse scored so that higher scores represented greater misinformation reliance across all items. As mentioned earlier, eight participants who responded inconsistently to normal versus reverse-coded items were excluded from analysis (see Supplement for details).

**Fig. 1:**
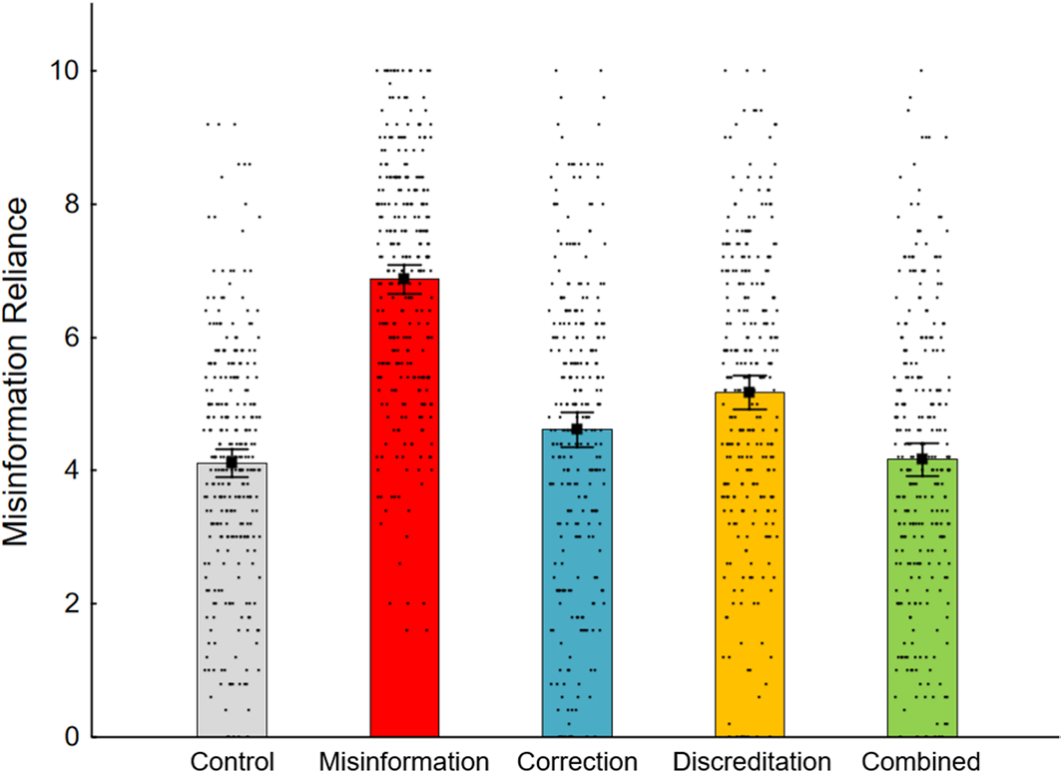
Mean misinformation reliance across conditions in Experiment 1. *Note.* Error bars show 95% confidence intervals

Misinformation reliance scores were computed by averaging responses to the inferential reasoning questions for each condition. Scores thus ranged from 0 to 10, with higher scores indicating stronger misinformation reliance. Results are shown in Fig. 1. A one-way repeated measures ANOVA (Huynh–Feldt corrected for sphericity violation) was conducted to compare misinformation reliance across conditions. This revealed a significant main effect of condition, *F*(4, 1164) = 95.85, *ε* = 0.97, *p* < 0.001, *η*_*p*_^2^ = 0.25. An additional 2 × 2 repeated measures ANOVA focussing on the experimental conditions with factors correction (no/yes) and discreditation (no/ yes) yielded main effects of correction, *F*(1, 291) = 154.25, *p* < 0.001, *η*_*p*_^2^ = 0.35, and discreditation, *F*(1, 291) = 85.32, *p* < 0.001, *η*_*p*_^2^ = 0.23, as well as a significant interaction, *F*(1, 291) = 29.76, *p* < 0.001, *η*_*p*_^2^ = 0.09. This showed that both a correction and a source discreditation had an effect and that a discreditation affected misinformation reliance differently depending on whether a correction was paired with it.

To test the hypotheses directly, planned contrasts were conducted, applying Holm–Bonferroni correction for multiple comparisons for each hypothesis-specific family of contrasts. Results are reported in Table 1. First, to establish whether the interventions were individually effective (H1), we tested whether correction and discreditation reduced misinformation reliance independently relative to the no-intervention misinformation condition. As predicted, misinformation reliance was higher in the misinformation condition (*M* = 6.87) compared to the correction (*M* = 4.62) or discreditation conditions (*M* = 5.17). Second, we tested whether the combination of interventions was more effective than their individual application (H2); the combined condition (*M* = 4.17) was contrasted with the discreditation and correction conditions. The combined intervention reduced misinformation reliance more than either correction or discreditation alone (note that the significant interaction in the 2 × 2 ANOVA mentioned earlier showed that the effect of the combined interventions was sub-additive, as predicted). Third, correction and discreditation conditions were contrasted to determine their relative efficacy (H3), revealing that the correction reduced misinformation reliance more than discreditation, as expected. Finally, the control condition (*M* = 4.11) was compared to each of the intervention conditions (correction, discreditation, and combined) to investigate whether there was a continued influence effect (i.e., significant misinformation reliance post-intervention; H4). This hypothesis was only partially supported: While the correction and discreditation conditions were significantly different from control, which is evidence of continued influence, the combined intervention eliminated misinformation reliance entirely.^3^

**Table 1:** Planned contrasts on misinformation reliance scores in Experiment 1

Hypothesis	Contrast	*F*(1, 291)	*η*_*p*_^2^	*P*
H1	Misinformation versus correction	157.13	0.531	< 0.001*
	Misinformation versus discreditation	110.07	0.274	< 0.001*
H2	Combined versus correction	7.62	0.026	0.006*
	Combined versus discreditation	36.12	0.110	< 0.001*
H3	Correction versus discreditation	9.61	0.032	0.002*
H4	Control versus correction	11.51	0.038	< 0.001*
	Control versus discreditation	45.90	0.136	< 0.001*
	Control versus combined	0.15	0.001	0.699

^*^Statistical significance post-Holm–Bonferroni adjustment

## Discussion

Experiment 1 tested if source discreditation is effective as an individual debunking strategy, and whether combining source discreditation with a correction can further reduce the continued influence of misinformation relative to the individual interventions. Results showed that both content-focused correction and source discreditation resulted in significant reductions in misinformation reliance, in line with Barnes et al. (2018). The observed efficacy of source discreditation demonstrates that individuals consider source characteristics when evaluating messages, consistent with research on the influence of source credibility on misinformation belief (Nadarevic et al., 2020; Swire et al., 2017; Zhu et al., 2010). The finding that the combination of correction and discreditation provided the most effective debunking corroborates recommendations for approaches that incorporate both strategies (MacFarlane et al., 2021; Paynter et al., 2019; Tay et al., 2022; Westbrook et al., 2023).

The heightened power of the combined approach may be explained by the simple fact that it provides a stronger intervention that targets both content and messenger. A discreditation might also make a correction more memorable or might reduce the psychological discomfort associated with a correction (Susmann & Wegener, 2022; Westbrook et al., 2023). Additionally, there may also be individual differences in who responds to various strategies. Speculatively, trait- or state-based differences in message versus source orientation may also be relevant in this context: Individuals attuned to information veracity may be more open to correction than discreditation, whereas individuals who strongly value honesty may respond more readily to discreditations. Alternatively, the observed sub-additivity may also be explained by a functional floor effect, given the use of Likert scales, which tend to show central tendency bias. As such, future research may consider alternative measures (e.g., open-ended questions).

Moreover, as predicted, corrections were found to be slightly more effective than discreditations, potentially because corrections more directly countered the misinformation in a way that was specifically relevant in terms of the inferential reasoning measure applied. It may also be the case that corrections are more effective because they tend to include subtle, implicit discreditation. However, this finding would need to be replicated with different materials before any strong conclusions can be drawn (cf. Barnes et al., 2018). Finally, while corrections and discreditations both resulted in some remnant misinformation reliance when compared to control (i.e., continued influence effects), the combined condition reduced misinformation reliance down to control levels. It is rare that debunking interventions are able to completely eliminate continued influence (e.g., see Ecker et al., 2010; Walter & Tukachinsky, 2020). In the current study, this was likely due to (1) the strength of the combined intervention and (2) the relatively high misinformation reliance in the control condition given the plausible nature of the misinformation used. Thus, there may be benefit in replication with open-ended questions (e.g., see Autry & Duarte, 2021; Prike et al., 2023a, 2023b) or less plausible materials (e.g., see Hinze et al., 2013).

In sum, Experiment 1 found discrediting a person source to be an effective intervention to reduce misinformation reliance. In real-world contexts, however, news reports are often provided by media sources. This is an important consideration, because the evidence regarding source-credibility effects in media contexts is overall less conclusive, leading some to argue that the credibility of media sources may have less impact on the acceptance of misinformation than the credibility of person sources (Ecker et al., 2022). Thus, it is unclear whether source discreditation is effective when the misinformation source is a media outlet as opposed to a person. The primary aim of Experiment 2 was to address this question.

## Experiment 2

The primary aim of Experiment 2 was to investigate whether discrediting a media source (as opposed to an individual source) can reduce subsequent misinformation influence. A secondary aim was to contrast discreditations that highlighted a conflict of interest with those that highlighted a track record of misleading communication. In the real world, discreditations may be more likely to rely on track-record information, as information regarding a specific conflict of interest is often unavailable. It was assumed that discreditations highlighting a poor track record may be less effective because they are less specific. It was further assumed that the superiority of highlighting a conflict of interest over a poor track record may be especially prominent for media sources. Media outlets may not be held to the same standards as people because they disseminate vast quantities of information, often by different authors, and often report on evolving events where information deemed correct at any given time may later turn out to be inaccurate (Lewandowsky et al., 2012; Porlezza & Russ-Mohl, 2012).

Participants read a series of fictional news articles, adapted from Experiment 1, containing a piece of misinformation originating from either a person or a media outlet. This misinformation was followed by a statement discrediting the misinformation source by indicating they had a conflict of interest or a history of making false claims. Two control conditions provided either just the misinformation with no discreditation (the misinformation condition) or neither the misinformation nor a discreditation (the no-misinformation control condition). As Experiment 2 contrasted person and media misinformation sources, an unspecified discreditation source was chosen so as not to introduce any asymmetry or added complexity to the design. Post-intervention reliance on misinformation was again assessed with inferential reasoning questions. Additionally, two questionnaires assessed perceived source credibility: one measured credibility of the misinformation source directly, while the other measured participants’ belief in new claims made by the misinformation source.

It was hypothesized that misinformation reliance and perceived source credibility would be reduced when the person or media outlet was discredited by pointing to a conflict of interest (H1a) or poor track record (H1b), relative to the misinformation condition. Secondly, it was predicted that the conflict-of-interest discreditation would be more effective at reducing misinformation reliance and source credibility than the track-record discreditation (H2). This prediction was made because a conflict of interest is a more specific reason to mistrust the source on the particular topic, whereas pointing to a poor track record may simply create the expectation that some claims from the relevant source may be false. Based on the above-mentioned assumption that people are held to higher standards than media outlets, the track-record discreditation was expected to be relatively less effective with a media source in particular, that is, an interaction was predicted such that the track-record discreditation would be more effective at reducing misinformation reliance and source credibility when the misinformation source was a person as opposed to a media outlet (H3). Finally, a secondary hypothesis was that there would generally be a continued influence effect for misinformation reliance in the conflict-of-interest (H4a) and track-record (H4b) discreditation conditions relative to the no-misinformation control (i.e., the interventions would not be fully effective).

## Method

Experiment 2 used a 4 × 2 mixed factorial design, with the within-subjects factor condition (no-misinformation control; misinformation; conflict-of-interest discreditation; track-record discreditation) and the between-subjects factor misinformation source (person; media outlet).

### Participants

In line with Experiment 1, a sample of 600 US-based Prolific users were recruited; the minimum platform approval rating was set to 97%. Based on a priori exclusion criteria, 11 participants were excluded for failing more than one attention check question, and 25 participants were excluded based on inconsistent responding between normal and reverse-coded items. An additional exclusion criterion of self-rated English proficiency as “poor” or “fair” on a 5-point Likert scale (1 = *poor* to 5 = *excellent*) did not apply to any participants. The final sample thus comprised *N* = 564 participants, who were randomly allocated to one of the two misinformation source groups (with the constraint of roughly equal group sizes): person (*n* = 281) and media outlet (*n* = 283). The sample included 299 males, 252 females, 11 non-binary participants, and two transgender men. Age range was 19–98 years (*M* = 44.19, *SD* = 14.59).

### Materials

An example article with all test questions is provided in Table 4; all articles and test questions can be found in the Supplement.

*Articles.* Three fictional news articles were adapted from Experiment 1 (topics: menopause treatment; new mine site; suburb burglary rate), and a new article was generated on a historic building (allegedly facing demolition due to safety concerns). There were eight versions of each article, defined by the 4 × 2 design. The no-misinformation control versions contained only generic information deemed to be factual. All other versions contained a piece of information deemed to be false that was attributed to either a person (e.g., “research fellow Dr Alana Rivett”) or a fictitious media outlet (e.g., “Frontier Magazine”). In the misinformation versions, this information was not challenged. In the discreditation versions, the misinformation was followed by a statement discrediting the misinformation source by indicating they either had a conflict of interest (e.g., they received funding from “Biotech Innovate, a pharmaceutical trade group heavily engaged in pharmaceutical lobbying”) or had a poor track record (e.g., they had a history of making “false, unfounded, and misleading claims”). The assignment of articles to conditions and the presentation order of articles and conditions were counterbalanced across participants using a Graeco-Latin square design; thus, participants in each misinformation source group were randomly allocated to one of four survey versions, and each participant read four articles—one per condition.

*Test questionnaires*. As an attention check to ensure adequate encoding, participants were required to respond to a set of four multiple-choice questions, one per article (e.g., “What was the topic of the 1st article you read?” a. Menopause; b. Depression; c. Acne; d. Hair loss). Additionally, participants completed three test questionnaires targeting inferential reasoning, source credibility, and new claim beliefs, respectively. Each questionnaire featured a set of questions relating to each article. All questions were presented on individual pages. The first questionnaire was identical to the questionnaire used in Experiment 1 but featured one additional inferential reasoning question per article (i.e., a total of six). The second questionnaire contained three questions regarding the credibility of the specific misinformation source presented in each article. Participants were asked to rate each source’s credibility as a source of information in the general domain of the misinformation claim (e.g., “How much would you *trust* what Dr Alana Rivett says about women’s health treatments in the future?”; 0 = *not at all* to 10 = *very much*). Across articles, the questions were identical except for the source and topic information provided. One question per article was reverse coded (e.g., “In future, Frontier Magazine should be considered an *unreliable* source of information on women’s health treatments”; 0 = *Strongly Disagree* to 10 = *Strongly Agree*). The final questionnaire presented three new claims per article, broadly related to the corresponding article’s misinformation claim, and asked participants to rate their belief in each claim, assuming it was made by the respective misinformation source (e.g., “If Frontier Magazine were to make the following claim, to what extent would you believe it? The new drug Tofasidone promises to be highly effective in reducing the symptoms of polycystic ovary syndrome”; 0 = *not at all* to 10 = *very much*). All questions are provided in the Supplement.

### Procedure

The procedure was similar to Experiment 1, with some key differences. First, participants were randomly assigned to one of the two misinformation source groups (person vs. media outlet) and then to one of the four corresponding survey versions. Immediately after reading all articles, participants completed the attention check questions, in the same order as the articles. Participants then completed the one-minute distractor task, after which they completed the inferential reasoning questionnaire. This was followed by the source-credibility and new claim questionnaires, which were presented in an interleaved fashion to optimize survey flow, that is, participants answered all remaining questions relating to each article (i.e., source-credibility and new claim questions regarding the first article) before proceeding to the next set of questions (i.e., source-credibility and new claim questions regarding the second article, and so on). Again, the order of the individual question sets within each questionnaire followed the order the articles were presented in. Participants received an additional prompt reminding them of the relevant article’s topic and misinformation source before each source-credibility questionnaire (e.g., “Please think back to the article on menopause and respond to the following questions about Frontier Magazine, which spoke about the search for menopause treatments”). Median completion time was 16 min; participants were paid £2.25 (approx. US$2.87).

## Results

After reverse scoring the responses to reverse-coded items, inconsistent responders on either inferential reasoning or source-credibility questionnaire were identified and excluded from analysis (*n* = 25; see Supplement for details).

### Misinformation reliance

**Fig. 2:**
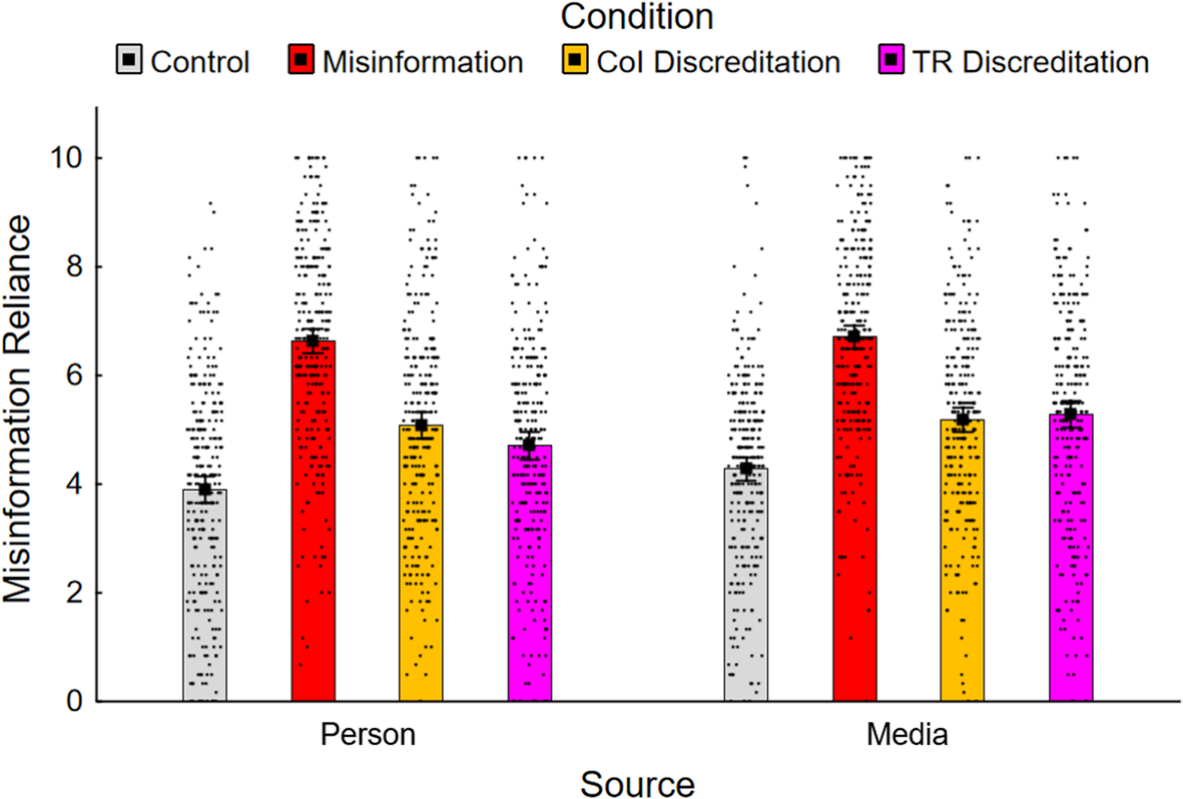
Mean misinformation reliance across conditions in Experiment 2. *Note.* CoI, conflict of interest; TR, track record. Error bars show 95% confidence intervals

Mean misinformation reliance scores were again calculated and are presented in Fig. 2. A 4 (condition: no-misinformation control, misinformation; conflict-of-interest discreditation; track-record discreditation) × 2 (misinformation source: person, media outlet) within-between ANOVA returned significant main effects of condition, *F*(3, 1686) = 171.53, *ε* = 0.98, *p* < 0.001, *η*_*p*_^2^ = 0.23, and misinformation source, *F*(1, 562) = 10.24, *p* = 0.001, *η*_*p*_^2^ = 0.02. There was no statistically significant interaction, *F*(3, 1686) = 2.27, *p* = 0.080.^4^

To directly test the specific hypotheses, a set of Holm–Bonferroni-corrected planned contrasts were conducted. The results are reported in the top panel of Table 2. First, to test whether the discreditation interventions were effective, each discreditation condition was contrasted with the misinformation condition for each misinformation source group. Both the conflict-of-interest (person, *M* = 5.09; media outlet, *M* = 5.18) and the track-record discreditations (person, *M* = 4.71; media outlet, *M* = 5.30) reduced misinformation reliance relative to the misinformation condition (person, *M* = 6.63; media outlet, *M* = 6.71), supporting H1a and H1b.

**Table 2:** Planned contrasts in Experiment 2

Hyp	Contrast	Group	*F*(1, 562)	*η*_*p*_^2^	*p*
*Misinformation reliance scores*					
H1a	Misinformation versus CoI Discred	Person	105.34	0.158	< 0.001*
		Media	103.64	0.156	< 0.001*
H1b	Misinformation versus TR Discred	Person	134.61	0.193	< 0.001*
		Media	72.95	0.115	< 0.001*
H2	CoI Discred. versus TR Discred	Person	5.21	0.009	0.023*
		Media	0.50	0.001	0.481
H3	Misinformation versus TR Discred	Person versus Media	4.80	0.008	0.029*
H4a	Control versus CoI Discred	Person	49.18	0.080	< 0.001*
		Media	28.48	0.048	< 0.001*
H4b	Control versus TR Discred	Person	26.65	0.045	< 0.001*
		Media	42.43	0.070	< 0.001*
*Source-credibility ratings*					
H1a	Misinformation versus CoI Discred	Person	189.09	0.252	< 0.001*
		Media	122.28	0.179	< 0.001*
H1b	Misinformation versus TR Discred	Person	289.96	0.340	< 0.001*
		Media	132.87	0.191	< 0.001*
H2	CoI Discred. versus TR Discred	Person	19.27	0.033	< 0.001*
		Media	1.10	0.002	0.295
H3	Misinformation versus TR Discred	Person versus Media	15.41	0.027	< 0.001*
*New claim belief ratings*					
H1a	Misinformation versus CoI Discred	Person	74.43	0.117	< 0.001*
		Media	67.08	0.107	< 0.001*
H1b	Misinformation versus TR Discred	Person	144.81	0.205	< 0.001*
		Media	75.72	0.119	< 0.001*
H2	CoI Discred. versus TR Discred	Person	12.79	0.022	< 0.001*
		Media	0.31	0.001	0.577
H3	Misinformation versus TR Discred	Person versus Media	5.67	0.010	0.018*

Hyp., hypothesis; CoI, conflict of interest; TR, track record; Discred., Discreditation

*Statistical significance after Holm–Bonferroni correction

Second, for each group, the relative efficacy of the discreditation interventions was investigated by contrasting conflict-of-interest and track-record discreditation conditions. In the person group, the contrast was significant, with misinformation reliance unexpectedly lower in the track-record discreditation condition. In the media outlet group, the difference was clearly non-significant. Accordingly, hypothesis H2 that the conflict-of-interest discreditation would be more effective than the track-record discreditation was rejected.

Third, an interaction contrast compared the misinformation and track-record discreditation conditions across groups to test if the efficacy of a track-record discreditation varied across misinformation sources. Consistent with H3, the interaction was significant; the track-record discreditation was more effective when the misinformation source was a person as opposed to a media outlet.

Finally, to test for a continued influence effect, planned contrasts were conducted to compare each discreditation condition against the no-misinformation control for each misinformation source group. Misinformation reliance was significantly greater in all discreditation conditions relative to control (person, *M* = 3.90; media outlet, *M* = 4.28); thus, a continued influence effect was present, and H4a and H4b were supported.

### Source credibility and new claim belief

**Fig. 3:**
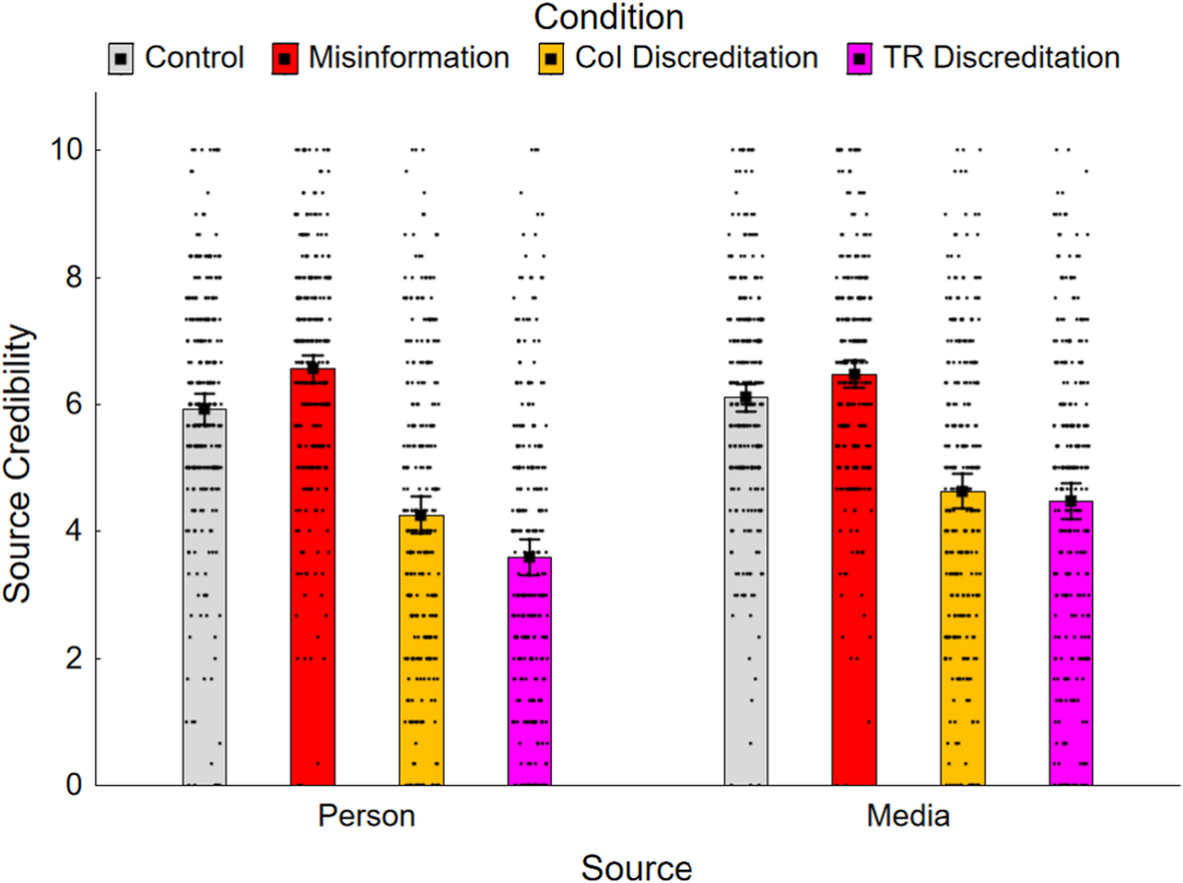
Mean source credibility across conditions in Experiment 2. *Note.* CoI, conflict of interest; TR, track record. Error bars show 95% confidence intervals

**Fig. 4:**
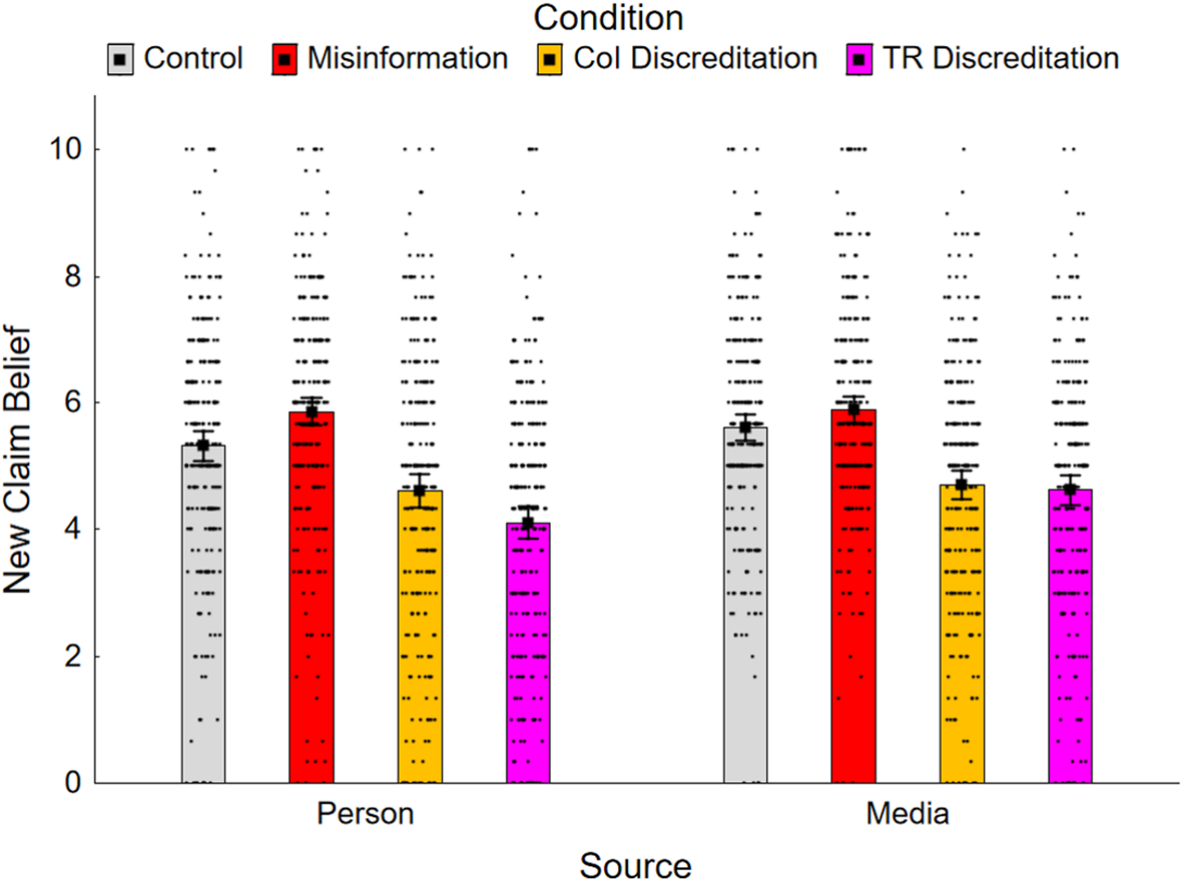
Mean new claim belief across conditions in Experiment 2. *Note.* CoI, conflict of interest; TR, track record. Error bars show 95% confidence intervals

Source-credibility and new claim belief ratings were calculated, for each condition, as the mean of the responses to the three respective questions. Both ratings ranged from 0 to 10, with higher scores indicating greater perceived credibility of the misinformation source. Results are shown in Figs. 3 and 4. Given similarity of measures and results, they are discussed jointly. A 4 (condition) × 2 (misinformation source) ANOVA on source-credibility ratings revealed significant main effects of condition, *F*(3, 1686) = 232.05, *ε* = 0.91, *p* < 0.001, *η*_*p*_^2^ = 0.292, and misinformation source, *F*(1, 562) = 7.80, *p* = 0.005, *η*_*p*_^2^ = 0.014, as well as a small but significant interaction, *F*(3, 1686) = 6.73, *p* < 0.001, *η*_*p*_^2^ = 0.012. This suggested that the impact of the discreditation on perceived source credibility was somewhat greater when the source was a person versus a media outlet, especially for track-record discreditations. With regard to new claim belief ratings, there was a significant main effect of condition, *F*(3, 1686) = 100.74, *ε* = 0.98, *p* < 0.001, *η*_*p*_^2^ = 0.152, and a marginal effect of misinformation source, *F*(1, 562) = 4.14, *p* = 0.042, *η*_*p*_^2^ = 0.01, again suggesting stronger discreditation impact for person versus media sources, but no significant interaction, *F*(3, 1686) = 2.50, *p* = 0.059.

The results of planned Holm–Bonferroni-corrected contrasts are reported in the lower two panels of Table 2. First, the effectiveness of each discreditation intervention was investigated by contrasting each discreditation condition against the misinformation condition for each misinformation source group. The conflict-of-interest discreditation condition was significantly lower than the misinformation conditions for both source-credibility ratings (person, *M* = 6.56 vs. *M* = 4.26; media outlet, *M* = 6.48 vs. *M* = 4.64) and new claim belief ratings (person, *M* = 5.86 vs. *M* = 4.60; media outlet, *M* = 5.89 vs. *M* = 4.70). The track-record discreditation conditions were also associated with significantly lower source-credibility ratings (person, *M* = 3.59; media outlet, *M* = 4.48) and new claim belief ratings (person, *M* = 4.10; media outlet, *M* = 4.63) relative to the misinformation conditions. Thus, consistent with H1a and H1b, source-credibility and new claim belief ratings were reduced in both discreditation conditions.

To compare the discreditation interventions, the conflict-of-interest discreditation condition was contrasted with the track-record discreditation condition for each misinformation source group. In the person group, source-credibility and new claim belief ratings were significantly lower in the track-record discreditation condition; in the media outlet group, there was no significant difference between the conditions. Therefore, H2—that ratings would be lower in the conflict-of-interest discreditation condition—was rejected.

Finally, to test the hypothesis that a track-record discreditation would have a stronger effect with a person versus media misinformation source, planned interaction contrasts compared the misinformation and track-record discreditation conditions across groups. Both contrasts were significant; thus, consistent with H4, the track-record discreditation was more effective in reducing source credibility and new claim beliefs if the misinformation source was a person rather than a media outlet.

## Discussion

Experiment 2 investigated whether the media is more resilient to discreditation than person sources and if highlighting a conflict of interest or a track record of misleading communication was a better source discreditation tool. Consistent with Experiment 1, discrediting either a person or media outlet reduced their perceived credibility (in line with Campos-Castillo & Shuster, 2021) and misinformation reliance (in line with Barnes et al., 2018). Given the inconclusive evidence from prior research regarding source-credibility effects in media contexts, it is useful to have obtained direct evidence that discreditations can be effective in reducing reliance on misinformation spread by a media outlet. It is possible that some of the conflicting findings in the literature resulted from methodological differences. For example, while the present study provided detailed and specific information relevant to the credibility of the misinformation source, other studies that found no effect of news source information simply enhanced the salience of source information (e.g., Dias et al., 2020) or provided abstract credibility ratings (e.g., Kim et al., 2019). Moreover, while the timing of the discreditations used in the current study required participants to retrospectively update their beliefs, it also made it likely that participants gave the discreditations undiluted attention. By contrast, source cues provided concurrently with a message, headline, or claim—as used in previous research—need to compete for attention. Future research could develop less-detailed discreditations that would allow for testing the effects of concurrent versus retrospective presentation in the same study. For instance, “conflict of interest” or “poor track-record” warnings could be displayed alongside versus following simulated social media posts.

The observed reduction in participants’ beliefs in new claims made by the discredited source suggests that discreditation effects can generalize to new claims and topics. In this respect, discreditations might be similar to inoculation interventions, which educate people about common misinformation tactics with the hope of reducing susceptibility to new misinformation encountered in the future (e.g., Lewandowsky & van der Linden, 2021).

It was further predicted that identifying a conflict of interest would be more impactful than pointing out a poor track record of communication because the former provides a specific reason to mistrust the source. However, the two types of intervention were found to be similarly effective (in fact, when discrediting a person, the track-record discreditation was slightly more effective). This is inconsistent with the notion that providing a motive for the dissemination of falsehoods is a particularly useful means to reduce perceived source credibility (Campos-Castillo & Shuster, 2021). However, the strength of any intervention is determined by various factors, and therefore, no firm general conclusions regarding relative efficacy can be drawn from one specific instantiation.

Moreover, as expected, track-record discreditations were more effective when the misinformation source was a person rather than a media outlet, corroborating the idea that media outlets may not be held to the same standards as people in this regard. Considering the sheer quantity of information media outlets publish, the time pressure they are under to do so, the variety of authors and editors at any given outlet, and the fact that media often report on evolving scenarios where information initially considered accurate may later turn out to be inaccurate (Lewandowsky et al., 2012; Porlezza & Russ-Mohl, 2012), the dissemination of misinformation may be perceived as less intentional, more excusable, and more variable. It should be noted, however, that the observed interaction effects were associated with small effect sizes and therefore may not be of much practical significance.

Finally, as predicted and in line with previous research, neither intervention entirely eliminated the continued influence of misinformation (Walter & Tukachinsky, 2020). The continued influence effects observed in this study might have resulted from the probabilistic nature of the discreditations, which may have contributed to uncertainty about whether or not the misinformation claim was indeed false (Connor Desai et al., 2020; Zmigrod et al., 2023). The fact that discreditations in Experiment 2 were not attributed to a specific source may have added to this uncertainty, although it is also possible that it may have enhanced demand effects, given that some participants may have perceived the experimenter as the discreditation source; however, given the close match of results across the two experiments, it seems unlikely that the lack of discreditation source in Experiment 2 had a marked effect.

## General discussion

In two experiments, this study provided evidence that source discreditations are an effective means to counter misinformation from both person and media sources. Experiment 1 demonstrated that discrediting a person source can reduce subsequent reliance on misinformation and can boost the efficacy of message-focused corrections. Experiment 2 replicated the effect of a stand-alone discreditation; pointing to either a conflict of interest or a poor track record of communication reduced subsequent reliance on misinformation from either person or media sources, and reduced perceived source credibility. Track-record discreditations were less effective for media sources, presumably because it is accepted that media outlets sometimes “get it wrong.”

At a theoretical level, these results substantiate suggestions that basic memory mechanisms alone cannot fully explain continued misinformation influence—failure to fully encode or retrieve corrective information can certainly contribute to continued influence effects, but clearly information evaluation plays a significant role as well. Therefore, models of continued influence need to account for additional factors, such as the impact of source characteristics on post-intervention misinformation reliance (e.g., Connor Desai & Reimers, 2023; Connor Desai et al., 2020; Ecker & Antonio, 2021; Ecker et al., 2022; O’Rear & Radvansky, 2020; Susmann & Wegener, 2022). This stance acknowledges that correcting misinformation presents a Bayesian inference problem in which the perceived reliability of the correction is weighed against the perceived reliability of the misinformation (Zmigrod et al., 2023). While these findings may not be overly surprising, it is important to rigorously test even predictions that may appear obvious, because it is certainly not always the case that intuitions are confirmed by empirical research.

A clear practical implication of the findings is that discrediting a misinformation source by attacking its credibility can be an effective debunking intervention. Further, as shown by the new claim belief findings of Experiment 2, highlighting a conflict of interest or a track record of inaccuracies may also provide benefits if a discredited source makes misleading claims in the future. However, potential ethical issues mean that discreditation interventions should be applied with caution. Specifically, the circumstances under which discrediting a source is justifiable must be carefully considered, particularly when the veracity of a claim being made is unknown. Discreditation is more justifiable if a source is a known and consistent purveyor of disinformation or conspiracy theories; in such cases, it may be more resource efficient to attack their credibility than to fact-check each individual claim they make (Harambam & Aupers, 2021; Lewandowsky et al., 2024). Despite proponents of epistemic relativism proclaiming that establishing objective ground truths is difficult or even impossible, scientific and historic (or event-related) facts do exist (Moberger, 2022). Therefore, if a person has a documented motivation to deny such facts, or a track record of doing so, it is arguably legitimate to point this out in the interest of open, candid, good faith debate, especially in situations where misleading claims have the potential to cause harm (Ecker et al., 2024; Jacques et al., 2008; Lewandowsky et al., 2024; Oreskes & Conway, 2010; Proctor, 2011; also see Tay et al., 2024).

Naturally, care needs to be taken during implementation to ensure that the discreditations themselves are based on verified information and delivered in an appropriate and targeted manner, as disparaging statements are likely to be ineffective (Wood et al., 2023). It would also be inappropriate to discredit generally reliable sources on the basis of infrequent or unintentional past errors; such behavior—which may also be used by disinformers to maliciously attack trustworthy information sources—should therefore be condemned. When message veracity is known, using source discreditations to bolster message-focused corrections (also see Westbrook et al., 2023) is perhaps more straightforwardly applied, as using multi-faceted interventions echoes existing recommendations (Ecker et al., 2022; Lewandowsky et al., 2020; Paynter et al., 2019). The Experiment 1 result that the combination was able to fully eliminate continued influence is promising in this regard.

Finally, several limitations must be noted. First and foremost, the current study purposefully used fictional materials to enhance experimental control and avoid topics that may be associated with strong prior attitudes. However, in real-world situations, people will have relevant pre-existing knowledge, beliefs, or attitudes toward the misinformation or its source, which may influence information processing (Susmann & Wegener, 2023a; Traberg & van der Linden, 2022; also see Ecker et al., 2022; Swire-Thompson et al., 2020). Thus, while this study provides proof of concept, findings are unlikely to generalize to all real-world situations in a straightforward manner. For example, discreditation efficacy will hinge on the discreditation source itself being viewed as at least somewhat credible. Moreover, in the real world, there can be a mismatch between source credibility and message veracity, that is, accurate information can come from a low-quality source, or a generally credible source can share incorrect information. Dias et al. (2020) suggested that such incongruence may interfere with a person’s ability to assess information veracity. Future studies should therefore explore how discreditations function in such mismatch situations. Additionally, future research could investigate potential effects on behavioral measures, as well as the temporal stability of discreditation effects (both in terms of post-intervention delays and delays between misinformation exposure and discreditation), which may be low due to reliance on potentially fragile source memory (also see Swire-Thompson et al., 2023; Tay et al., 2023).

## Conclusion

In conclusion, these experiments offer compelling evidence that discrediting sources can effectively counter misinformation originating from both individuals and media outlets. Additionally, effective source discreditation can be achieved by either highlighting a specific conflict of interest or pointing to a track record of making false claims. This supports using source discreditation, either alone or in combination, as a strategy for combating misinformation, reducing the perceived credibility of dubious sources, and reducing reliance on new false claims those sources make in the future.

## Data Availability

Materials and datasets generated and analyzed during the current study are available in the OSF repository at https://osf.io/vqth3/. We note that for both experiments, we have reported all measures, conditions, data exclusions, and how sample sizes were determined.

## Appendix: Example articles and test questions from both experiments

See Tables 3 and 4.

**Table 3:** Example article and test questions, Experiment 1

Information	Text
Generic introductory information	Menopause is the natural cessation of a woman’s menstrual cycle. Many women report discomfort related to menopausal symptoms such as hot flashes, insomnia, dry skin, and weight gain. A commonly prescribed treatment is hormonal replacement therapy, such as estrogenic creams and tablets “With aging often comes disruption to our bodily systems and processes, particularly when women reach menopausal age,” says women’s health researcher Dr Alana Rivett. “Symptoms can be quite debilitating, so we are always looking for new treatments.”
Misinformation	*“Quite often we find that specific interventions can be used in the treatment of different conditions. That’s how we discovered that a drug called Cendexyl, initially developed to treat low blood pressure, is also an effective treatment for menopause symptoms.”*
Correction	*However, according to gynecologist Dr Katherine Lyons, it is incorrect that Cendexyl is an effective treatment for menopause symptoms. “Based on the overall evidence available, this is just entirely false. Claims promoting Cendexyl for menopause treatment should therefore be disregarded.”*
Discreditation	*However, according to gynecologist Dr Katherine Lyons, Dr Rivett has a conflict of interest as she did not disclose that her research is funded by Pharmarix, the pharmaceutical company behind Cendexyl. “Dr Rivett’s claims promoting Cendexyl to treat menopause should therefore be disregarded.”*
Combined correction and discreditation	*However, according to gynecologist Dr Katherine Lyons, it is incorrect that Cendexyl is an effective treatment for menopause symptoms. “Based on the overall evidence available, this is just entirely false.” Further, Lyons states that Dr Rivett has a conflict of interest; she did not disclose that her research is funded by Pharmarix, the pharmaceutical company behind Cendexyl. “Dr Rivett’s claims promoting Cendexyl to treat menopause should therefore be disregarded.”*
Generic concluding information	As well as medical approaches, people can reduce some menopause symptoms through lifestyle changes like regular exercise, eating a balanced diet, and improving sleep hygiene. To understand the best way to manage your health, please seek the advice of your doctor
Inferential reasoning questions	(1) “From Insomnia to Hot Flashes: Blood Pressure Meds Help with Menopause” would be an appropriate headline for this report. [Strongly Disagree (0)–Strongly Agree (10)] (2) Cendexyl treatments should be covered by health insurance for menopausal symptoms. [Strongly Disagree (0)–Strongly Agree (10)] (3) Doctors should refrain from prescribing Cendexyl for menopause. [Strongly Disagree (0)–Strongly Agree (10)] (R) (4) I would ***not*** recommend Cendexyl to a relative or friend going through menopause. [Strongly Disagree (0)–Strongly Agree (10)] (R) (5) How effective do you think Cendexyl is in the treatment of menopause symptoms? [Entirely Ineffective (0)–Very Effective (10)]

Non-italicized text constitutes the no-misinformation control version; all other versions included this text. The italicized text was specific to the misinformation, correction, discreditation, and combined versions and is labeled accordingly. All correction and discreditation versions included the misinformation text. (R), reverse-coded test questions

**Table 4:** Example article and test questions, Experiment 2

Information	Text
Generic introductory information	With aging often comes disruption to our bodily systems and processes, particularly when women reach menopausal age. Menopause is the natural cessation of a woman’s menstrual cycle Many women report discomfort related to menopausal symptoms such as hot flashes, insomnia, dry skin, and weight gain. A commonly prescribed treatment is hormonal replacement therapy, such as estrogenic creams and tablets. Symptoms can be quite debilitating, so pharmacologists are always looking for new treatments
Misinformation-Only—Person	*Quite often, specific interventions can be used in the treatment of different conditions. For example, research fellow Dr Alana Rivett stated that “a drug called Cendexyl, initially developed to treat low blood pressure, has also been found to be an effective treatment for menopause symptoms.”*
Misinformation-Only—Media	*Quite often, specific interventions can be used in the treatment of different conditions. For example, Frontier Magazine recently reported that “a drug called Cendexyl, initially developed to treat low blood pressure, has also been found to be an effective treatment for menopause symptoms.”*
CoI Discreditation—Person	*However, it has been pointed out that Dr Rivett has a conflict of interest; she receives funding from Biotech Innovate, a pharmaceutical trade group heavily engaged in pharmaceutical lobbying. This calls into question Dr Rivett’s claims promoting Cendexyl to treat menopause*
CoI Discreditation—Media	*However, it has been pointed out that Frontier Magazine has a conflict of interest; the magazine is partially funded by Biotech Innovate, a pharmaceutical trade group heavily engaged in pharmaceutical lobbying. This calls into question Frontier Magazine’s claims promoting Cendexyl to treat menopause*
TR Discreditation—Person	*However, it has been indicated that Dr Rivett has a history of making false, unfounded, and misleading claims. This raises doubts about Dr Rivett’s claims promoting Cendexyl to treat menopause*
TR Discreditation—Media	*However, it has been indicated that Frontier Magazine has a history of publishing false, unfounded, and misleading claims. This raises doubts about Frontier Magazine’s claims promoting Cendexyl to treat menopause*
Generic concluding information	As well as medical approaches, people can reduce some menopause symptoms through lifestyle changes like regular exercise, eating a balanced diet, and improving sleep hygiene. To understand the best way to manage your health, please seek the advice of your doctor
Inferential reasoning questions	(1) “From Insomnia to Hot Flashes: Blood Pressure Meds Help with Menopause” would be an appropriate headline for this report. [Strongly Disagree (0)–Strongly Agree (10)] (2) Cendexyl treatments should be covered by health insurance for menopausal symptoms. [Strongly Disagree (0)–Strongly Agree (10)] (3) Doctors should refrain from prescribing Cendexyl for menopause. [Strongly Disagree (0)–Strongly Agree (10)] (R) (4) I would ***not*** recommend Cendexyl to a relative or friend going through menopause. [Strongly Disagree (0)–Strongly Agree (10)] (R) (5) It would be fine to market Cendexyl as a drug for menopause symptoms. [Strongly Disagree (0)–Strongly Agree (10)] (6) How likely do you think it is that Cendexyl is effective in the treatment of menopause symptoms? [Very Unlikely (0)–Very Likely (10)]
Source-credibility questions	(1) How much would you **trust** what *SOURCE* says about women’s health treatments in the future? [Not At All (0)–Very Much (10)] (2) How **credible** do you think *SOURCE* is generally as a source of information on women’s health treatments? [Not At All (0)–Very Much (10)] (3) In future, *SOURCE* should be considered an **unreliable** source of information on women’s health treatments. [Strongly Disagree (0)–Strongly Agree (10)] (R)
New claim belief questions	(1) The new drug Tofasidone promises to be highly effective in reducing the symptoms of polycystic ovary syndrome. [Not At All (0)–Very Much (10)] (2) Clinical trials demonstrate no currently available treatment shows satisfactory reduction in melasma, a skin condition affecting approx. 2% of adult women in the US. [Not At All (0)–Very Much (10)] (3) The side effects of drugs commonly used to delay premature labor in pregnant women have been vastly overstated. [Not At All (0)–Very Much (10)]

Non-italicized text constitutes the no-misinformation control version; all other versions included this text. The italicized text was specific to the misinformation and discreditation versions and is labeled accordingly. All discreditation versions included the misinformation text. CoI, conflict of interest; TR, track record; (R), reverse-coded test questions; *SOURCE*, Dr Alana Rivett (person); Frontier Magazine (media outlet). New claim belief questions were prefaced by “If *SOURCE* were to make the following claim, to what extent would you believe it?”

## Footnotes

1 In part, the effect was likely also driven by the presence of explicit credibility signals in the environment leading participants to generally consider post-veracity more thoroughly (in a manner similar to accuracy nudges, e.g., see Butler et al., 2024; Pennycook et al., 2020).

2 Note that all hypotheses relate to a difference between two conditions; the power analysis therefore applies to all hypotheses.

3 Supplementary analyses including survey version as a factor showed that while there were slight differences in how the interventions affected misinformation reliance scores across various scenarios, there was reasonable consistency, with even the poorest performing correction and discreditation significantly reducing misinformation reliance when compared to no-intervention (see Supplement for details).

4 Supplementary analyses including survey version as a factor showed that while there were slight differences in how the interventions affected misinformation-reliance scores across various scenarios, there was reasonable consistency, with even the poorest performing discreditations significantly reducing misinformation reliance when compared to no-intervention (see Supplement for details).
